# Circulating MicroRNA Profiling Identifies Distinct MicroRNA Signatures in Acute Ischemic Stroke and Transient Ischemic Attack Patients

**DOI:** 10.3390/ijms24010108

**Published:** 2022-12-21

**Authors:** Salman M. Toor, Eman K. Aldous, Aijaz Parray, Naveed Akhtar, Yasser Al-Sarraj, Essam M. Abdelalim, Abdelilah Arredouani, Omar El-Agnaf, Paul J. Thornalley, Sajitha V. Pananchikkal, Ghulam Jeelani Pir, Raheem Ayadathil, Ashfaq Shuaib, Nehad M. Alajez, Omar M. E. Albagha

**Affiliations:** 1 College of Health and Life Sciences (CHLS), Hamad Bin Khalifa University (HBKU), Qatar Foundation (QF), Doha P.O. Box 34110, Qatar; 2 Diabetes Research Center, Qatar Biomedical Research Institute (QBRI), Hamad Bin Khalifa University (HBKU), Qatar Foundation (QF), Doha P.O. Box 34110, Qatar; 3 The Neuroscience Institute, Academic Health System, Hamad Medical Corporation (HMC), Doha P.O. Box 3050, Qatar; 4 Qatar Genome Program, Qatar Foundation Research, Development and Innovation, Qatar Foundation (QF), Doha P.O. Box 5825, Qatar; 5 Neurological Disorders Research Center, Qatar Biomedical Research Institute (QBRI), Hamad Bin Khalifa University (HBKU), Qatar Foundation (QF), Doha P.O. Box 34110, Qatar; 6 Division of Neurology, Department of Medicine, University of Alberta, Edmonton, AB T6G 2R3, Canada; 7 Department of Neurology, Hamad Medical Corporation (HMC), Doha P.O. Box 3050, Qatar; 8 Translational Cancer and Immunity Center, Qatar Biomedical Research Institute (QBRI), Hamad Bin Khalifa University (HBKU), Qatar Foundation (QF), Doha P.O. Box 34110, Qatar; 9 Rheumatology and Bone Disease Unit, Centre for Genomic and Experimental Medicine, Institute of Genetics and Cancer, University of Edinburgh, Edinburgh EH4 2XU, UK

**Keywords:** microRNA, miRNome, ischemic, stroke, acute ischemic stroke, transient ischemic attack

## Abstract

Transient ischemic attack (TIA) refers to a momentary neurologic deficit caused by focal cerebral, spinal or retinal ischemic insult. TIA is associated with a high risk of impending acute ischemic stroke (AIS), a neurologic dysfunction characterized by focal cerebral, spinal or retinal infarction. Understanding the differences in molecular pathways in AIS and TIA has merit for deciphering the underlying cause for neuronal deficits with long-term effects and high risks of morbidity and mortality. In this study, we performed comprehensive investigations into the circulating microRNA (miRNA) profiles of AIS (*n* = 191) and TIA (*n* = 61) patients. We performed RNA-Seq on serum samples collected within 24 hrs of clinical diagnosis and randomly divided the study populations into discovery and validation cohorts. We identified a panel of 11 differentially regulated miRNAs at FDR < 0.05. Hsa-miR-548c-5p, -20a-5p, -18a-5p, -484, -652-3p, -486-3p, -24-3p, -181a-5p and -222-3p were upregulated, while hsa-miR-500a-3p and -206 were downregulated in AIS patients compared to TIA patients. We also probed the previously validated gene targets of our identified miRNA panel to highlight the molecular pathways affected in AIS. Moreover, we developed a multivariate classifier with potential utilization as a discriminative biomarker for AIS and TIA patients. The underlying molecular pathways in AIS compared to TIA may be explored further in functional studies for therapeutic targeting in clinical translation.

## 1. Introduction

Stroke is the second leading cause of global mortality and the third leading cause of death and disability combined, responsible for an estimated 6.5 million deaths in 2019 [1]. It is described as a neurological deficit attributed to acute focal injury to the central nervous system (CNS) due to impaired blood perfusion and is broadly classified as ischemic, hemorrhagic or subarachnoid [2]. Ischemic strokes refer to incident neurological dysfunction due to acute cerebral, spinal or retinal infarction by ischemic insult [2] and constitute the majority (62.4%) of all incident stroke cases [1]. Stroke diagnosis and management require clinical investigations and brain imaging to assess the extent of neuronal damage and to determine reperfusion therapies.

Transient ischemic attack (TIA) refers to focal neurological deficits that resolve within minutes to 24 h after onset, and imaging reveals no acute stroke in the majority of cases. Stroke risk is high (~7.5–17.4%) in the initial days following a TIA [3,4,5,6]. TIA occurs in ~20% of ischemic stroke patients in the short time preceding stroke [7]. The risk of myocardial infarction and nonvascular mortality is also high in both TIA and stroke cases [8]. Thus, TIA requires immediate diagnosis and management to mitigate stroke risk. However, a significant proportion of TIA cases remain underdiagnosed due to lack of reporting [9]. The fundamental difference between TIA and AIS is the lack of acute infarction in most patients with TIA. The lack of residual incident neuronal deficits and absence of sustained clinical presentations in TIA hinders the risk stratification for the development of subsequent AIS. Understanding the differences in molecular pathways in AIS and TIA has merit for deciphering the underlying cause of neuronal deficits with long-term risks of morbidity and mortality.

MicroRNAs (miRNAs) are non-coding RNA molecules, which can regulate the expression of specific gene targets by affecting target mRNA stability or repressing translational efficiency. These molecules are detectable in circulation and are recurrently explored as predictive, diagnostic or prognostic biomarkers for various physiological conditions [10]. Investigating the disturbances in miRNA profile (miRNome) can decipher the physiological and molecular disturbances in various diseases, including cardiovascular disease [11]. Several groups have investigated circulating miRNAs in ischemic strokes [12]. However, these studies have predominantly utilized targeted approaches using microarrays to identify distinct circulating miRNAs in AIS patients or excluded other types of strokes including TIAs. Next-generation sequencing (NGS) technologies have emerged as a robust technique in miRNA expression profiling and combination with in silico tools can enhance their utilization as blood-based biomarkers due to high detection sensitivity and accuracy [13]. However, the few studies that have utilized next-generation sequencing technologies for miRNA profiling of stroke patients were limited by the modest patient sample size [14,15,16,17]. Thus, comprehensive miRNA profiling of AIS and TIA patients is warranted for the robust identification of differentially regulated miRNAs, which may be explored as discriminative biomarkers for AIS and TIA.

We have previously reported a panel of 10 differentially regulated circulating miRNAs to distinguish between AIS patients and healthy individuals [18], and a panel of 5 circulating miRNAs between AIS patients with type 2 diabetes mellitus (T2DM), a known risk factor for stroke that is also associated with worse disease outcomes, compared to non-diabetic AIS patients [19]. In the present study, we compared the circulating miRNA profiles of clinically diagnosed AIS and TIA patients using serum samples for identifying differentially regulated circulating miRNAs. We also probed the molecular pathways associated with the experimentally validated gene targets of these miRNAs and investigated the clinical pathology endpoints and diseases associated with disruptions in these pathways. Importantly, we developed a classifier based on our identified panel of dysregulated miRNAs to differentiate between AIS and TIA cases as a potential low-invasive miRNA-based biomarker. The underlying molecular pathways in AIS compared to TIA may be explored further in functional studies for therapeutic targeting and clinical translation.

## 2. Results

### 2.1. Identification of Differentially Regulated Circulating miRNAs in AIS Patients Compared to TIA Patients

**Figure 1:**
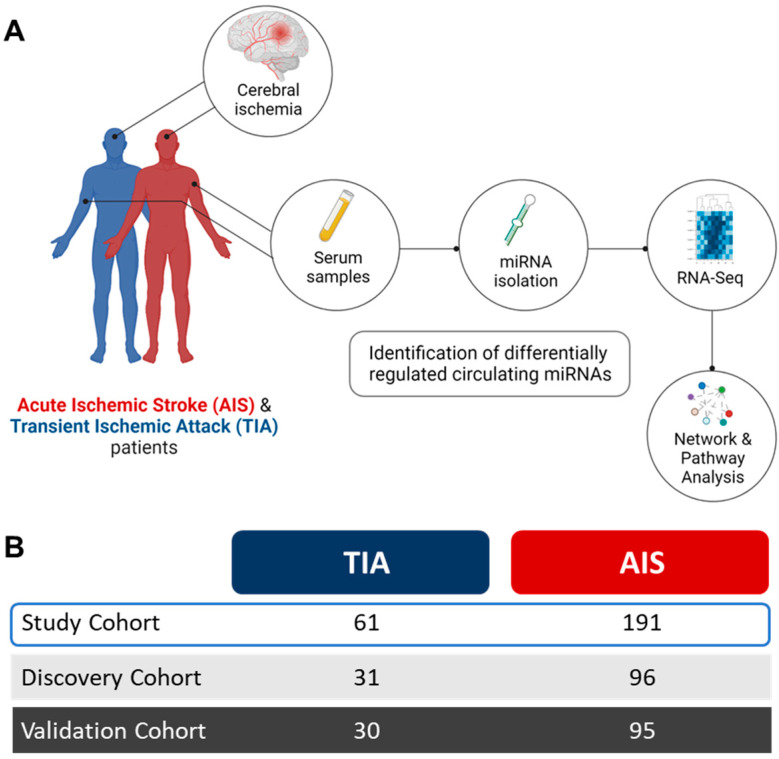
Study design and analysis workflow. (**A**). Serum samples were collected from acute ischemic stroke (AIS) and transient ischemic attack (TIA) patients within 24 h of clinical diagnosis, to purify circulating microRNAs (miRNAs) for transcriptomic analysis by RNA-Seq. Stringent analyses criteria enabled identification of differentially regulated circulating miRNAs in AIS patients compared to TIA patients. Downstream analyses for the gene targets of identified miRNA panel were performed to highlight the molecular pathways and networks affected in AIS. (**B**). The study population comprised TIA (*n* = 61) and AIS (*n* = 191) patients who were randomly divided into discovery and validation cohorts for identification of replicated, differentially regulated circulating miRNAs in AIS patients compared to TIA patients.

Serum samples from AIS and TIA patients were analyzed for the identification of distinct circulating miRNAs. The study design and analysis workflow are depicted in Figure 1 and the characteristic features of study population are listed in Table 1.

**Figure 2:**
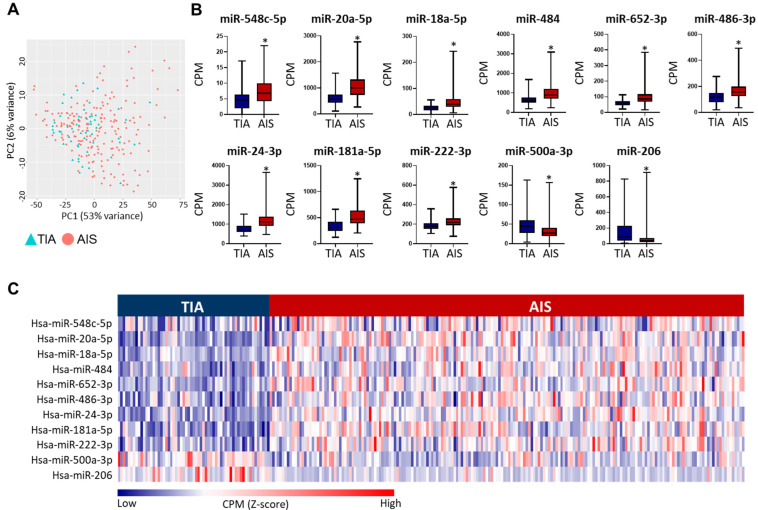
Circulating miRNA profiles of acute ischemic stroke (AIS) and transient ischemic attack (TIA) patients. (**A**). PCA plot depicts the variability in miRNA expression between TIA and AIS patients. (**B).** Box and whiskers plots show the differences in counts per million (CPM) in AIS and TIA patients of the 11 differentially regulated, validated miRNAs. Mean with minimum and maximum values, and upper and lower quartiles are depicted for each data set with significant comparisons annotated by an asterisk (*) on top (*p* < 0.0001). (**C**). Heatmap shows the dysregulation in identified miRNA panel (Z-scores calculated CPM) between AIS and TIA patients in the overall study population.

The principal component analysis (PCA) of the miRNA profiles of AIS and TIA patients did not show any distinct clustering of the two cohorts (Figure 2A). The differential expression analyses for miRNA transcripts of AIS and TIA patients were compared in the discovery and validation datasets separately to identify the statistically significant (FDR < 0.05), differentially regulated circulating miRNAs that were replicated in both analyses. In the discovery dataset, 24 miRNAs showed varying degrees of fold change (FC) and fulfilled the analyses criteria (FDR < 0.05, Supplementary Table S1). Out of these, 11 miRNAs were replicated in the validation cohort by meeting the analysis cutoff (Table 2). Moreover, the datasets showed the same direction of effect in the discovery and validation sets for all 11 miRNAs (Table 2). Out of the 11 miRNAs, 9 miRNAs were upregulated and 2 were downregulated in AIS compared to TIA patients (Figure 2B,C). Overall, this panel of 11 miRNAs is presented as differentially regulated miRNAs in AIS versus TIA patients.

In addition, we divided AIS patients into two groups based on the National Institutes of Health Stroke Scale (NIHSS) scores; minor to moderate stroke (NIHSS score: 0–5, *n* = 152) and severe stroke (NIHSS score: ≥6, *n* = 39), and compared the expression levels of our identified miRNA panel. We found that only hsa-miR-652-3p showed dysregulation in AIS patients with severe stroke, but this was with borderline significance (fold change = −1.23, FDR = 0.05). Moreover, we also found that 2 miRNAs out of our identified miRNA panel also showed significant dysregulation in AIS patients compared to healthy controls (Supplementary Table S2), based on our previously reported datasets comparing AIS patients with healthy controls [18].

### 2.2. Predictive Capacity of the Identified miRNA Panel in AIS Patients

**Figure 3:**
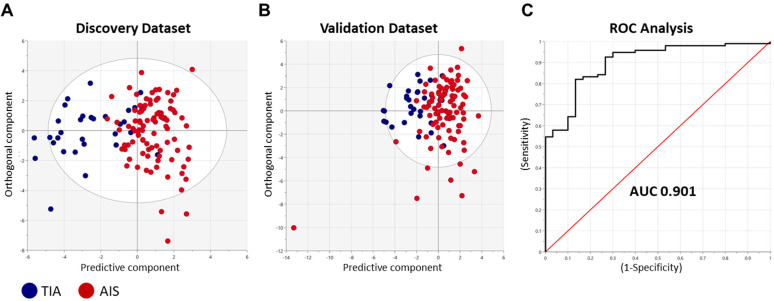
Discriminative performance of the identified miRNA panel to identify AIS patients. Orthogonal projections to latent structures-discriminant analysis (OPLS-DA) was performed using the top differentially regulated miRNAs (*n* = 24) in the discovery cohort data. The classifier was trained on data from all participants in (**A**). discovery cohort (*n* = 122) and tested on the (**B**). validation cohort (*n* = 119). Scatter plots show the predictive component to discriminate AIS cases from TIA cases (blue dots—*x*-axis) versus the orthogonal component representing a multivariate confounding effect that is independent of stroke (maroon dots—*y*-axis). (**C**). ROC curve analysis generated an area under the curve (AUC) of 0.901.

To determine the predictive performance of our identified miRNA panel to distinguish between AIS and TIA patients, we performed multivariate discriminant analyses (Figure 3). The orthogonal projections to latent structures discriminant analysis (OPLS-DA) method was adopted using the top FDR-significant differentially regulated miRNAs (*n* = 24, Supplementary Table S1) identified from the discovery cohort data (Figure 3A) and testing the classifier model on the validation cohort (Figure 3B) to determine its performance. The classifier showed high predictive performance, evidenced by an AUC value of 0.901 generated on the ROC curve analysis (Figure 3C).

### 2.3. Identification of Molecular Pathways Modulated by Circulating miRNAs in AIS Patients

**Figure 4:**
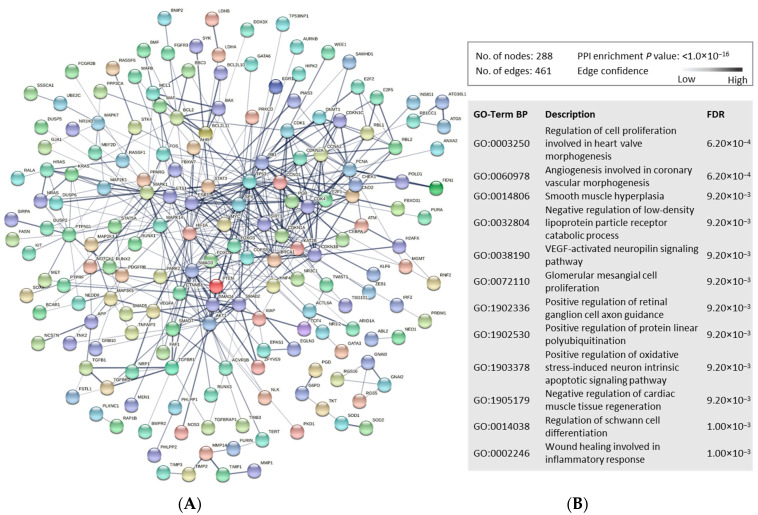
Functional enrichment analysis of the proteins encoded by the gene targets of dysregulated miRNAs in AIS versus TIA patients. (**A**). The protein–protein interaction (PPI) network generated for the 345 gene targets (unique genes; *n* = 292, protein coding genes; *n* = 288) of the identified miRNA panel is shown. Network nodes represent proteins, while edges depict protein–protein associations. They key network statistics are also presented. (**B**). The top functional enrichment annotations from Gene Ontology (GO) Biological Process are listed.

miRNAs can regulate the gene expression of multiple genes, thereby affecting the molecular function of their transcribed proteins. To decipher the potential molecular pathways affected in AIS compared to TIA, we first identified the known gene targets (experimentally validated with strong evidence) of our miRNA panel (Total *n* = 345 genes, *n* = 252 unique genes; Supplementary Table S3). We then generated protein–protein interaction (PPI) networks using STRING tool to explore and annotate pathways associated with proteins encoded by the gene targets of our identified miRNA panel (Figure 4). The PPI network showed high enrichment and associations of multiple proteins in mediating several pathways. Gene Ontology (Biological Processes; GO-Term BP) annotations predominantly corresponded to cardiac vascularity and neuronal cell functionality annotations, alongside protein processing and other cellular processes (Figure 4).

### 2.4. Cellular Processes and Clinical Pathology Endpoints Associated with Gene Targets of the Dysregulated miRNAs in AIS Patients

**Figure 5:**
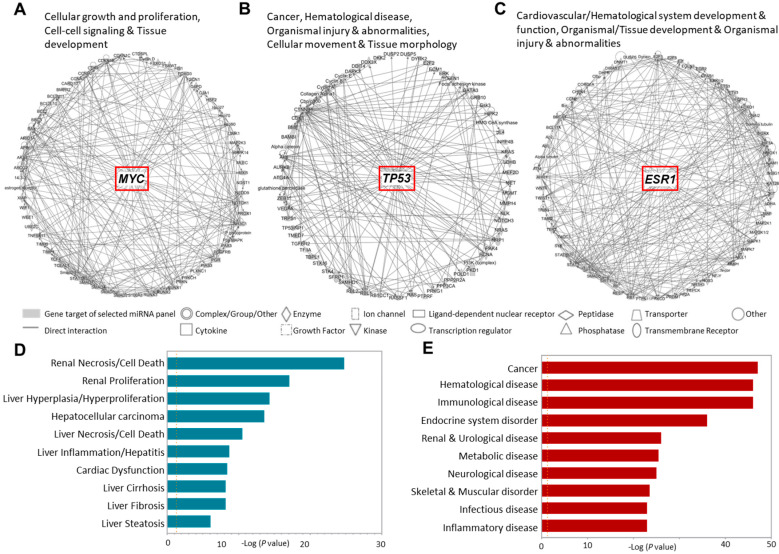
Disease annotation and Ingenuity pathway analysis for the gene targets of dysregulated miRNAs in AIS versus TIA patients. The gene targets of the 11 dysregulated miRNAs in AIS compared to TIA patients were analyzed for disease/function annotation and network analysis. (**A**–**C**). Ingenuity pathway network analysis of the gene targets of dysregulated miRNAs in AIS patients are shown. (**D**–**E**). Bar plots show the clinical pathology endpoints and diseases annotations associated with these genes.

Identifying the molecular and cellular pathways that contribute to the underlying pathologies leading to the high mortality associated with AIS is crucial for therapeutic intervention. We investigated the molecular networks that involved the gene targets of our identified dysregulated miRNA panel in AIS patients (Figure 5A–C). We found that our genes of interest are intensely involved in cellular growth and interactions in tissue development, mediated by MYC activation (Figure 5A). In addition, we found the involvement of these genes in the aberrant molecular networks mediated by p53 and ESR1 in cancer; hematological development and pathologies; organismal injuries that include edema, hemorrhage and lesions; and cellular chemotaxis associated with tissue morphology and development (Figure 5B,C).

Identifying the clinical pathology endpoints associated with deviations in these molecular networks showed associations with hepatorenal and cardiac pathologies (Figure 5D). In addition, a broad spectrum of diseases covering cancer, hematological, neurological, immunological, endocrinological and metabolic disorders were associated with deviancies in these networks (Figure 5E).

## 3. Discussion

We investigated the differences in circulating miRNA regulatory network of AIS and TIA patients to highlight the potential molecular pathways affected in AIS patients. We identified a panel of 11 differentially regulated miRNAs in AIS compared to TIA patients using stringent criteria. Many of the identified miRNAs are broadly involved in cerebrovascular integrity or function via neuroprotection or in promoting neuroinflammation. Previous studies have reported dysregulation of many of these miRNAs in various cerebrovascular disorders and suggested them as disease biomarkers [20,21,22] or associated variants of their gene targets with stroke [23] or cardiac diseases [24].

The dysregulation of hsa-miR-548c-5p has been previously associated with intracranial aneurysm [25] and hypertension [26]. Moreover, its downregulation in plasma was associated with venous thrombosis [27]. These findings indicate the roles of miR-548c-5p in the modulation of circulation and vascularity, but it has not been directly linked with stroke. Moreover, numerous putative gene targets of miR-548c-5p have been predicted but none have been experimentally validated till present. Our novel findings of upregulation of miR-548c-5p in AIS patients as one of the most significantly dysregulated miRNAs highlights its relevance to the pathogenesis of stroke, which warrants further investigations.

In agreement with our findings, the upregulation of circulating miR-20a-5p, miR-18a-5p and miR-181a-5p has been previously reported in AIS patients compared to healthy controls [28]. MiR-20a-5p has been presented as a discriminatory biomarker between embolic and thrombotic strokes [29]. Notably, one of the common validated gene targets of miR-20a-5p and miR-206, *VEGFA* has been previously associated with stroke severity. Circulating VEGF levels were higher in ischemic stroke patients and showed prognostic significance [30]. Moreover, serum VEGF levels were associated with severe disability in AIS patients [31]. In addition, genome-wide association studies (GWAS) have associated variants in *VEGFA* with coronary heart disease [32] and myocardial infarction [24], and variants in other gene targets of miR-20a-5p, *CDKN1A* (also a common gene target of miR-181a-5p) [23] and *PRKG1* [33] with stroke.

Hsa-miR-18a-5p promotes the differentiation of vascular smooth muscle cells [34] and has been reported as a biomarker for venous malformation [35]. Serum hsa-miR-18a-5p levels have also been associated with cardiovascular anomalies and proposed as a circulating biomarker for atherosclerosis [22]. The protein encoded by one of its gene targets, CTGF (also known as *CCN2*) is over-expressed in atherosclerotic plaques [36]. CTGF levels were higher in stroke than TIA and are associated with plaque stabilization following stroke [37]. Moreover, GWAS have associated *CTGF* with coronary artery calcification in type 2 diabetes patients [38]. Upregulation of miR-18a-5p in AIS patients indicates its potential roles in thrombotic or embolic ischemic strokes but needs further investigation.

The upregulation of miR-484 was reported in patients with hyperacute cerebral infarction [39] and its dysregulation has been reported in patients with ruptured intracranial aneurysms compared to those with intact intracranial aneurysm, along with miR-486-3p, miR-181a-5p, miR-222-3p and miR-500a-3p [25]. MiR-652-3p was significantly upregulated in freshly resected brain tissues of stroke patients with severe infarction and life-threatening cerebral swelling, compared to control temporal lobe samples [40]. Notably, the shared gene target of miR-484 and miR-652-3p, *ZEB1* was previously explored in relation to ischemic neuronal injury. ZEB1 was induced in response to cerebral ischemia and is involved in neuronal cell survival [41], while its upregulation in microglia associated with alleviating post-AIS cerebral injury by reducing neuroinflammation [42].

The dysregulation of miR-486-3p has been associated with aneurysmal subarachnoid hemorrhage [43], while miR-24-3p has been associated with delayed cerebral vasospasm, which occurs in around one-third of aneurysmal subarachnoid hemorrhage patients [44]. Among the gene targets of miR-486-3p, *FASN* has been reported as a critical mediator in de novo lipogenesis in astrocytes following cerebral ischemic injury [45], while *SYK* is involved in cerebral inflammatory activation after ischemic stroke [46]. The upregulation of miR-24-3p in AIS patients concurs with previous reports of its upregulation as exosomal miRNA in AIS patients [47]. Additionally, the neuroprotective role of its gene target *ACVR1B* in promoting remyelination has been reported previously in a mouse model of ischemic stroke [48]. Importantly, GWAS have associated variants of several of the gene targets of miR-24-3p;*FAF1* [49]*, GATA3* [50]*, MMP14* [51]*, NOS3* and *PTPRF* [23] with stroke.

Hsa-miR-181a-5p is associated with inflammation, upregulated in response to TGFβ signaling [52], and has been identified as a critical regulatory miRNA in ischemic strokes [53]. Serum levels of the protein encoded by its gene targets *ATG5* is presented as a biomarker for AIS patients [54], while another gene target, ATM is involved in neuroprotection in the initiation of protective mechanisms in ischemic preconditioning but promotes neuronal cell death following the ischemic insult [55]. Additionally, upregulation of miR-222-3p was previously observed in acute myocardial infarction patients [56]. Moreover, its gene target *AR1D1A* associated with cerebral lesions of white matter hyperintensities in stroke patients in GWAS [57].

Several gene targets have been predicted for miR-500a-3p, but none experimentally validated. Our findings of its significant downregulation in AIS patients compared to TIA patients suggest its regulation by post-stroke neuroprotective mechanisms but further investigations are warranted to explore its role in the pathophysiology of stroke. In contrast, the levels of miR-206 were higher in cardioembolic stroke patients with hemorrhagic transformation [58]. Moreover, in line with our findings, the downregulation of miR-206 in circulation of AIS patients has been reported previously and was also associated with diagnostic significance [20].

The proteins encoded by the gene targets of our identified miRNA panel showed strong interactions indicating the pathophysiological processes, which may be disrupted in AIS patients compared to TIA patients. These networks showed strong annotations with processes involved in cardiac vascularity and muscle regeneration, neuronal differentiation and apoptosis, angiogenic pathways with possible involvement in wound healing, and catabolic protein biochemical processing. Collectively, these processes highlight the underlying protein pathways responsible for long-term effects and clinical complications of AIS compared to TIA patients. In addition, deciphering the aberrant molecular pathways in AIS patients showed homeostasis-associated processes in tissue maintenance and functionality. In relation to AIS, we found that disruptions in these pathways are associated with hepatorenal and cardiac anomalies, and vast diseases covering metabolic, inflammatory, endocrine and immunological disorders, which may be involved in morbidity and mortality associated with AIS.

Uncovering the pathophysiological and molecular imbalances in stroke has potentials of improving the understanding of the etiopathogenesis of the cerebrovascular neurological events with high risk of permanent neurologic disability and mortality. Tissue infarction due to cerebral ischemia triggers necrotic and apoptotic neuronal cell death. The ischemic core in the brain experiences irreversible insult and necrotic cell death, while the cells in ischemic penumbra undergo apoptosis via mitochondria-mediated intrinsic mechanisms, and cell death receptor-mediated extrinsic mechanisms of apoptosis [59,60]. Additional mechanisms of post-ischemic neuronal damage include necroptosis, autophagy and excitotoxicity [61]. We report that our identified dysregulated miRNAs target several critical mediators of cell death, most notably P53, Bcl-2, tumor necrosis factor family proteins, protein kinases such as Akt, and autophagy-related (ATG) proteins, among others. These findings indicate the involvement of the processes involved in neuronal cell death and may be investigated further in relation to AIS.

Our panel of dysregulated miRNAs showed high discriminative performance in assessing AIS and TIA patients, which shows promising clinical utilization. Importantly, despite identification of several candidate biomarkers, TIA remains a clinical diagnosis and development of robust diagnostic protocols is necessitated [62]. Moreover, the current individual blood-based biomarkers for AIS diagnosis also lack robust performance for use in clinical settings [63]. Notably, while these biomarkers predominantly discriminate between TIA or AIS and healthy controls, our identified miRNA panel distinguishes between AIS and TIA patients and benefits more from superior discriminatory performance than candidate biomarkers for stroke, such as C-reactive protein levels (AUC 0.73 [64,65]). However, further experimental validations for confirming expression are required. Moreover, we highlighted the crucial pathways potentially disrupted or exploited in AIS, showed potential utilization of differentially regulated circulating miRNAs as discriminatory biomarkers for AIS and TIA patients and identified several gene targets and pathways, which may be investigated further. However, the lack of confirmation of miRNA/gene/protein expression, functional validation, modest sample size of TIA patients and absence of validation in an external dataset necessitate additional investigations. Overall, the remarkably distinct circulating miRNA profiles of AIS patients compared to TIA patients strengthens the rationale for further research into their clinical application as low-invasive disease biomarkers.

## 4. Materials and Methods

### 4.1. Study Population

The study population (*n* = 252) comprised clinically diagnosed acute ischemic stroke (AIS; *n* = 191) and transient ischemic attack (TIA; *n* = 61) patients admitted to Hamad General Hospital (Doha, Qatar). All patients provided written informed consent prior to sample donation. This study was approved by the institutional review boards of Qatar Biomedical Research Institute, Doha, Qatar (Protocol no. 2019-013) and Hamad Medical Corporation (Protocol no. 15304/15), Doha, Qatar. Patients’ medical records were also accessed to retrieve relevant information. Characteristic features of the study population are listed in Table 1. AIS patients were also divided into two groups based on the clinical severity of stroke as assessed on the NIHSS scale.

### 4.2. Study Design

Fresh serum samples (200 µL) were collected from AIS and TIA patients within 24 h of the cerebrovascular event and stored at −80 °C for subsequent miRNA profiling by RNA-Seq. The study population was divided randomly into discovery and validation cohorts with proportionate distribution of covariates (age, gender, prior administration of DM management and cholesterol lowering drugs).

The miRNA profiles of AIS patients (*n* = 96) were first compared with TIA patients (*n* = 31) in the discovery cohort. The differential expression analyses were performed while adjusting for age, gender, diabetes and statin treatment to identify significant (false discovery rate (FDR) < 0.05) miRNAs in the discovery cohort. Similar analysis was performed on the validation cohort (AIS; *n* = 95, TIA; *n* = 30) to identify the FDR-significant miRNAs, which were replicated from analyses of the discovery cohort. These miRNAs were presented as the panel of differentially regulated miRNAs in AIS versus TIA patients. A metanalysis for the overall datasets was also performed to confirm our findings. The study design and analysis workflow are depicted in Figure 1. Downstream analyses to explore the potential pathways affected by the panel of dysregulated miRNAs in AIS patients were also performed (explained in succeeding sections).

### 4.3. miRNA Isolation and Sequencing

The miRNA transcripts from serum samples were sequenced as described previously [18]. Briefly, miRNeasy Serum/Plasma Advanced Kit (Qiagen, Hilden, Germany) was used to purify total RNA from serum samples. RNA quantification was carried out using Qubit RNA Broad Range Assay Kit (Invitrogen, Carlsbad, CA, USA). The libraries were prepared using QIAseq miRNA next-generation sequencing (NGS) Library Kit (Qiagen) using QIAseq miRNA NGS 96 Index IL kit (Qiagen) for indexing. The quality-passed libraries, assessed using Qubit dsDNA HS assay kit (Invitrogen) and Agilent 2100 Bioanalyzer DNA1000 chip (Agilent Technologies, Santa Clara, CA, USA), were pooled using TruSeq PE Cluster Kit v3-cBot-HS kit (illumina, San Diego, CA, United States). HiSeq 3000/4000 SBS kit (illumina) was used to perform sequencing on illumina HiSeq 4000 system (10 million reads per sample).

### 4.4. Data Curation and Analyses

The sequencing data were generated as single reads (at 75 cycles) and alignment was carried out on CLC Genomics Workbench (v.21.0.5, Qiagen) utilizing the human miRbase v22 reference genome. miRNA transcript expression levels were computed as counts per million (CPM) from the total counts of mapped miRNA reads. Calibration for RNA spike-in (RNA transcript of known sequence and quantity) was also performed. The differential expression analyses were carried out on RStudio (version 4.1.1; RStudio, Boston, MA, USA) utilizing the DSEq2 method [66], while adjusting for covariates (age, gender, DM and statin treatment). Statistical analyses and representations were performed using GraphPad Prism 9.1.2 (GraphPad Software, Columbia, ML, USA). A *p* value of <0.05 was considered statistically significant.

### 4.5. Discriminant Analyses

Discriminant analysis and classification was performed to determine the predictive performance of our identified panel of miRNAs to discriminate between AIS and TIA patients. The orthogonal projections to latent structures discriminant analysis (OPLS-DA) classifier was first trained on the findings of the discovery datasets (statistically significant differentially regulated miRNAs in AIS versus TIA patients) using SIMCA multivariate data analysis software (version 16; Umetrics, Vasterbottens Lan, Sweden). The model was then tested on the validation dataset and the performance was assessed by determining the area under the curve (AUC) value of the receiver operating characteristic (ROC) curve.

### 4.6. Downstream Pathway and Network Analyses

The previously identified gene targets with strong evidence of experimental validation of the identified miRNA panel dysregulated in AIS patients compared to TIA patients were retrieved from the miRTargetLink 2.0 database [67]. Target gene enrichment and protein–protein interaction (PPI) network analysis was performed by STRING [68]. The functional pathway network analyses were performed on QIAGEN Ingenuity Pathway Analysis (IPA) software (QIAGEN Inc., https://digitalinsights.qiagen.com/IPA (accessed on 12 September 2022)) [69].

## Figures and Tables

**Table 1:** Characteristic features of the study cohort.

Characteristic	Discovery Cohort		Validation Cohort	
	TIA ^†^	AIS ^‡^	TIA	AIS
Number	31	96	30	95
Age	48.23 ± 9.68	50.38 ± 9.40	49.43 ± 10.84	50.01 ± 9.50
Gender (Male/Female)	28/3	86/10	26/4	90/5
Diabetes mellitus (DM) (no/yes)	19/12	49/47	17/13	50/45
Hypertension (no/yes)	19/12	26/70 *	13/17	25/70 *
Smoking (no/yes)	14/17	56/40	19/11	51/44
DM medication (no/yes)	23/8	73/23	22/8	66/29
Statin medication (no/yes)	22/9	83/13 *	21/9	78/17
Total cholesterol (mmol/L)	4.16 ± 0.96	4.85 ± 1.22 *	4.54 ± 0.73	5.16 ± 1.20 *
LDL-C (mmol/L)	2.65 ± 1.04	3.15 ± 1.08	2.84 ± 0.82	3.34 ± 1.12 *
Triacyl glycerides (mmol/L)	1.74 ± 0.63	1.54 ± 0.81	1.38 ± 0.60	1.90 ± 1.09 *

^†^ Transient ischemic attack. ^‡^ Acute ischemic stroke. * Statistically significant compared to TIA.

**Table 2:** Differentially regulated miRNAs in AIS versus TIA patients.

	Discovery		Validation		Combined	
miRNA	FC *	FDR **	FC	FDR	FC	FDR
hsa-miR-548c-5p	1.80	2.44 × 10^−2^	1.90	1.56 × 10^−2^	1.70	1.13 × 10^−4^
hsa-miR-20a-5p	1.69	1.52 × 10^−7^	1.73	1.19 × 10^−4^	1.69	1.72 × 10^−14^
hsa-miR-18a-5p	1.55	2.29 × 10^−2^	1.73	1.48 × 10^−2^	1.72	5.01 × 10^−6^
hsa-miR-484	1.52	1.08 × 10^−3^	1.55	8.32 × 10^−3^	1.47	2.64 × 10^−6^
hsa-miR-652-3p	1.49	4.76 × 10^−3^	1.62	1.08 × 10^−3^	1.53	2.31 × 10^−7^
hsa-miR-486-3p	1.46	3.93 × 10^−3^	1.49	9.51 × 10^−3^	1.46	5.73 × 10^−7^
hsa-miR-24-3p	1.45	3.93 × 10^−3^	1.63	6.79 × 10^−4^	1.48	1.13 × 10^−7^
hsa-miR-181a-5p	1.45	4.25 × 10^−3^	1.61	1.19 × 10^−4^	1.44	2.44 × 10^−7^
hsa-miR-222-3p	1.19	4.46 × 10^−2^	1.34	3.85 × 10^−3^	1.24	1.39 × 10^−5^
hsa-miR-500a-3p	−1.67	1.41 × 10^−2^	−1.65	4.02 × 10^−2^	−1.63	8.05 × 10^−5^
hsa-miR-206	−3.18	3.92 × 10^−4^	−2.39	2.57 × 10^−2^	−3.20	2.95 × 10^−9^

* Fold change. ** False discovery rate.

## Data Availability

All data generated or analyzed during this study are included in this published article (and its Supplementary Information files) or are available from the corresponding author by reasonable request.

## Supplementary Materials

The supporting information can be downloaded at: https://www.mdpi.com/article/10.3390/ijms24010108/s1.
