# Supplementary material for: Circulating MicroRNA Profiling Identifies Distinct MicroRNA Signatures in Acute Ischemic Stroke and Transient Ischemic Attack Patients
Source: Int J Mol Sci. 2022 Dec 21;24(1):108. doi: 10.3390/ijms24010108 (PMC9820644; doi:10.3390/ijms24010108)
Supplement: Supplementary file 1 [file ijms-24-00108-s001.zip › Supplementary_Table_S1.pdf]

Supplementary Table S1. Top statistically significant (FDR<0.05) differentially regulated miRNAs in AIS versus TIA patients in the discovery cohort.

miRNA	FC*	FDR**
hsa-miR-3158-3p	1.89	$6.81x10^{-3}$
hsa-miR-548c-5p	1.80	$2.44x10^{-2}$
hsa-miR-132-3p	1.72	$4.42x10^{-3}$
hsa-miR-20a-5p	1.69	$1.52x10^{-7}$
hsa-miR-18a-5p	1.55	$2.29x10^{-2}$
hsa-miR-484	1.52	$1.08x10^{-3}$
hsa-miR-652-3p	1.49	$4.76x10^{-3}$
hsa-miR-486-3p	1.46	$3.93x10^{-3}$
hsa-miR-24-3p	1.45	$3.93x10^{-3}$
hsa-miR-181a-5p	1.45	$4.25x10^{-3}$
hsa-miR-374a-5p	1.45	$4.08x10^{-2}$
hsa-miR-451a	1.43	$4.68x10^{-3}$
hsa-miR-92a-3p	1.41	$5.18x10^{-4}$
hsa-miR-32-5p	1.38	$2.29x10^{-2}$
hsa-miR-363-3p	1.38	$6.81x10^{-3}$
hsa-miR-361-5p	1.37	$6.81x10^{-3}$
hsa-miR-130a-3p	1.33	$1.94x10^{-2}$
hsa-miR-222-3p	1.19	$4.46x10^{-2}$
hsa-miR-664a-5p	-1.34	$4.47x10^{-2}$
hsa-miR-500a-3p	-1.67	$1.41x10^{-2}$
hsa-miR-30e-3p	-1.76	$1.59x10^{-4}$
hsa-miR-342-5p	-2.06	$1.19x10^{-2}$
hsa-miR-206	-3.18	$3.92x10^{-4}$
hsa-miR-184	-3.70	$4.25x10^{-3}$
hsa-miR-3158-3p	1.89	$6.81x10^{-3}$

*Fold change. **False Discovery Rate.
