# Supplementary material for: Circulating MicroRNA Profiling Identifies Distinct MicroRNA Signatures in Acute Ischemic Stroke and Transient Ischemic Attack Patients
Source: Int J Mol Sci. 2022 Dec 21;24(1):108. doi: 10.3390/ijms24010108 (PMC9820644; doi:10.3390/ijms24010108)
Supplement: Supplementary file 1 [file ijms-24-00108-s001.zip › Supplementary_Table_S2.pdf]

Supplementary Table S2. Common statistically significant (FDR<0.05) differentially regulated miRNAs in AIS versus TIA patients and Healthy Controls.

	AIS† vs TIA‡		AIS vs HC♦	
**miRNA**	**FC***	**FDR****	**FC**	**FDR**
hsa-miR-486-3p	1.46	5.73×10^{-7}	2.94	9.56×10^{-58}
hsa-miR-206	-3.20	2.95×10^{-9}	-3.33	3.86×10^{-14}

†Acute Ischemic Stroke. ‡Transient Ischemic Attack. ♦Healthy Controls. *Fold change. **False Discovery Rate.
