# Supplementary material for: Circulating MicroRNA Profiling Identifies Distinct MicroRNA Signatures in Acute Ischemic Stroke and Transient Ischemic Attack Patients
Source: Int J Mol Sci. 2022 Dec 21;24(1):108. doi: 10.3390/ijms24010108 (PMC9820644; doi:10.3390/ijms24010108)
Supplement: Supplementary file 1 [file ijms-24-00108-s001.zip › Supplementary_Table_S3.pdf]

Supplementary Table S3. Gene targets of the identified miRNA panel dysregulated in AIS patients compared to TIA patients.

miRNA	Target
hsa-miR-548c-5p	*
hsa-miR-20a-5p	*ABL2, ANKH, APP, ARHGAP12, ATG16L1, BAMBI, BCL2, BCL2L11, BMPR2, BNIP2, CCND1, CCND2, CDKN1A, CRIM1, DAPK3, DNMT1, DUSP2, E2F1, EGLN3, EGR2, EPAS1, ETV1, FBXO31, GJA1, HIF1A, IRF2, ITGB8, KIF26B, KIT, LIMK1, MAP2K3, AP3K12, MAP3K5, MCL1, MEF2D, MYC, NFKBIB, PHLPP2, PKD1, PKNOX1, PPARG, PPP2R2A, PRKG1, PTEN, PTPRO, PURA, RB1, RB1CC1, RBL1, RBL2, REST, RGS5, RUNX1, RUNX3, SIRPA, SMAD4, SMAD7, STAT3, TCEAL1, TGFBR1, TGFBR2, TIMP2, TP53INP1, TSG101, UBE2C, VEGFA, WEE1, ZFYVE9*
hsa-miR-18a-5p	*ATM, BCL2, BCL2L10, CDK19, CTGF, DICER1, DNMT1, ESR1, FCGR2B, HIF1A, HSF2, IRF2, MEF2D, NEDD9, NEO1, NR1I2, NR3C1, PHLPP1, PIAS3, PTEN, RUNX1, SDC4, SMAD2, SMAD3, SMAD4, STK4, TBPL1, TGFBR2, TNFAIP3, TNFSF11*
hsa-miR-484	*FIS1, SMAD2, ZEB1*
hsa-miR-652-3p	*LLGL1, ZEB1*
hsa-miR-486-3p	*BCL11A, ECM1, FASN, MAF, PRKCD, SYK*
hsa-miR-24-3p	*ABCB9,ACVR1B, AGPAT2, ARHGAP19, ATG4A, AURKB, BCAR1, BCL2L11, BRCA1, CARD10, CCNA2, CCND1, CDK1, CDK4, CDKN1B, CDKN2A, CHEK1, COPS5, CORO1A, CYP11B2, DEDD, DHFR, DHFRP1, DND1, DYRK2, E2F2, EIF2S3, FAF1, FBXW7, FEN1, FGF11, FGFR3, FSCN1, FURIN, GATA3, H2AFX, HMOX1, HNF4A, IFNG, IFNR, IL4, INSIG1, JPH2, LDHA, LDHB, MAFB, MAP3K9, MAPK14, MAPK7, MEN1, MLEC, MMP14, MT1M, MXI1, MYC, NCAN, NCSTN, NDST1, NOS3, NOTCH1, PAK4, PCNA, PDGFRB, POLD1, PRDX6, PRKCH, PSAP, PTPN9, PTPRF, REG4, S100A8, SH3PXD2A, SLC6A4, SP1, SSSCA1, ST7L, STX16, TACC3, TGFB1, TMED7, TMEM92, TNK2, TP53, TRIB3, TRIM11, WNT4, XIAP, ZNF217*
hsa-miR-181a-5p	*ABCG2, AHR, ATG5, ATM, BAX, BCL2, BCL2L11, CDKN1A,CDKN1B, CDX2, CEBPA, COL16A1, CTDSPL, CTNNB1, DDIT4, DDX3X, DUSP5, DUSP6, E2F5, EGR1, FOS, GATA6, GPD1L, GPR78, HIPK2, HRAS, IFNG, INPP4B, KAT2B, KLF6, KRAS, MAP2K1, MAPK1, MCL1, MEG3, MTMR3, NLK, NOTCH1, NRAS, PBX3, PGR, PHLPP2, PLAG1, PPP3CA, PRAP1, PRKCD, PRKN, PROX1, PTEN, PTPN11, PTPN22, RALA, RAP1B, RASSF1, RASSF6, RGS16, RGS5, RNF2, RUNX1, SAMHD1, SIRT1, STAT3, TCF4, TERT, TGFBR1, TGFBRAP1, TIMP1, TUSC3, TWIST1, WIF1, XIAP, ZNF763*
hsa-miR-222-3p	*ABCG2, ADAM1A, ARID1A, BBC3, BMF, CDKN1B, CDKN1C, CERS2, CORO1A, DICER1, DIRAS3, DKK2, ESR1, ETS1, FOS, FOXO1, FOXO3, GAS5, GJA1, GNAI2, GNAI3, GRB10, ICAM1, KIT, MGMT, MMP1, PLXNC1, PPP2R2A, PRDM1, PTEN, RECK, RUNX2, SMAD5, SOD2, SSSCA1, SSX2IP, STAT5A, TCEAL1, TIMP3, TMED7, TNFSF10, TRPS1, VGLL4*
hsa-miR-500a-3p	*
hsa-miR-206	*ACTL6A, AKT1, ANXA2, CCL2, CCND1, CCND2, CDK4, ESR1, FSTL1, G6PD, GJA1, GPD2, KRAS, MET, NOTCH3, NR1H3, NR4A2, NRP1, OTX2, PAX3, PGD, SFRP1, SMAD2, SOD1, SOX9, TAC1, TBX3, TKT, TWF1, TWIST1, UTRN, VAMP2, VEGFA*

*No experimentally validated gene target.
